# P-907. Burden of Hospitalization for Coccidioidal Meningitis: A National Cohort Study

**DOI:** 10.1093/ofid/ofae631.1098

**Published:** 2025-01-29

**Authors:** Craig I Coleman, Fariba Donovan, Lahar Miriyapalli, Ryan M Shan, Mark Bresnik, Belinda Lovelace

**Affiliations:** University of Connecticut, Storrs, Connecticut; University of Arizona, Valley Fever Center for Excellence, Tucson, Arizona; University of Connecticut School of Pharmacy, Monroe, New York; University of Connecticut School of Pharmacy, Monroe, New York; F2G, Ltd., Princeton, New Jersey; F2G, Inc., Princeton, New Jersey

## Abstract

**Background:**

Coccidioidal meningitis (CM) is a severe complication of an endemic fungal infection requiring life-long aggressive medical and often surgical management necessitating hospitalization. Few data evaluating the burden of CM on hospitals have been published. This report assesses inpatient healthcare utilization, total costs, and mortality in hospitalized CM patients.

**Table 1:**
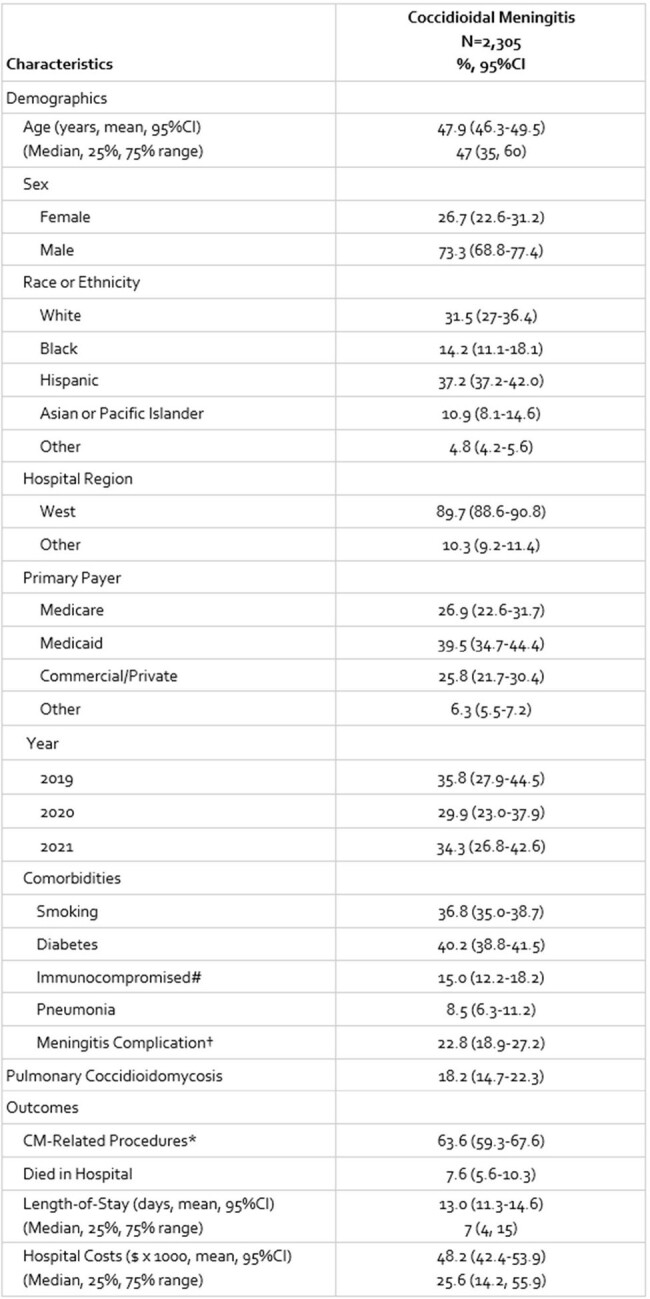
Demographics, Comorbidities and Outcomes of Patients Admitted with Coccidioidal Meningitis CI=confidence intervals; CM=coccidioidal meningitis †Primary diagnosis code for a common meningitis complication including hydrocephalus, cerebral vascular accident, encephalitis, encephalopathy, cerebral vasculitis or vasospasm, cerebral abscess, shunt complication, infusion device failure. *Includes ventricular shunt, drain or infusion device placement, revision, or

removal.

**Methods:**

We used the National Inpatient Sample (NIS) to evaluate CM hospitalizations for the years 2019 t0 2021. The NIS is a 20% stratified systematic randomized sampling of all hospital discharges, drawn from States participating in the Healthcare Utilization Project (HCUP) (including all states in which coccidioidomycosis is endemic) and covering >97% of the US population. The sample weighted to reflect all hospitalizations in the US per year. CM was identified by the International Classification of Diseases-10th Revision (ICD-10) diagnosis code of B38.4 in any coding position. All analyses were performed on the weighted population using methods prescribed by HCUP. We estimated frequencies or mean/median values for demographics, comorbidities, need for CM-related procedures (i.e., ventricular shunt, drain or infusion device placement, revision, or removal), inpatient mortality, length-of-stay (LOS), and all cause total hospital costs (in 2023 US$) with 95% confidence intervals (CIs).

**Results:**

We identified 2,305 CM hospitalizations (**Table 1**). The annual rates of CM hospitalizations ranged from 2.7-3.2 per million Americans for the three years studied. The mean age of CM patients was ∼48 years, most were men (73.3%) and occurred in the West census region (89.7%). Concurrent pulmonary coccidioidomycosis was present in 18.2% of patients. In total, 63.6% (59.3-67.6%) of patients required a CM-related procedure during the stay and 7.6% (5.6-10.3%) died prior to discharge. Mean LOS was 13.0 days (11.3-14.6) and mean hospital costs were $48,155 ($42,382-$53,929) per stay.

**Conclusion:**

Hospitalization of CM patients in the US are associated with substantial medical and economic burden. The complex management and high costs associated with CM suggest a need for novel management approaches including novel antifungal treatments.

**Disclosures:**

**Craig I. Coleman, PharmD**, F2G Ltd: Advisor/Consultant|F2G Ltd: Grant/Research Support **Fariba Donovan, MD, PhD**, F2G: Advisor/Consultant|F2G Ltd: Advisor/Consultant|F2G Ltd: Grant/Research Support|F2G Ltd: Honoraria **Mark Bresnik, MD**, F2G Ltd: Employee **Belinda Lovelace, PharmD, MS, MJ**, F2G, Inc.: Employee

